# Florida Dental Law

**Published:** 1887-09

**Authors:** 


ARTICLE VI.

### FLORIDA DENTAL LAW.

Following is the text of the bill entitled "an act to provide for the appointment of a board of examiners of dentists, and to regulate the practice of dentistry in the State of Florida," which has been passed by the Legislature of the State and become a law:

*Be it enacted by the Legislature of the State of Florida:*

SECTION I. That from and after the passage of this act it shall be unlawful for any person to engage in the practice of dentistry in the State of Florida unless said person shall have obtained a certificate from a board of dentists, duly authorized and appointed under the provisions of this chapter to issue license.

SEC. 2. Be it further enacted, That the board of examiners shall consist of five (5) dental graduates or practitioners of dentistry, appointed by the Governor, and who are members in good standing of the Florida State Dental Association; provided, that said graduates or practitioners have been practicing in the State of Floridr for a term of not less than three (3) years. Said board shall be appointed to serve two (2) years. The president of said board shall have power to fill all vacancies in said board for unexpired terms.

SEC. 3. Be it further enacted, That it shall be the duty of this board: First, to meet annually at the time of the meeting of the Florida State Dental Association, or oftener at the call of any three members of said board; thirty days' notice must be given of the annual meeting; secondly to prescribe a course of reading for those who study dentistry under private instructors; thirdly, to grant license to all applicants who undergo a satisfactory examination; fourthly, to keep a book in which shall be registered the names of all persons licensed by said board to practice dentistry in the State of Florida.

SEC. 4. Be it further enacted, That three members of said board shall constitute a quorum for the transaction of business, and should a quorum not be present on the day appointed for their meeting, those present may adjourn from day to day until a quorum is present.

SEC. 5. Be it further enacted, That one member of said board shall have the power and may grant a license to an applicant to practice dentistry until the next regular meeting of said board, when he shall report the fact, at which time the temporary certificates shall expire, but such temporary certificates shall not be granted by a member of the board after the board has rejected the applicant.

SEC. 6. Be it further enacted, That any person who shall, in violation of this act, practice dentistry in the State of Florida, shall be deemed guilty of a misdemeanor, and upon conviction shall be punished by a fine of not less than twenty-five dollars nor more than five hundred dollars; provided, that nothing in this act shall be construed so as to prevent any person from extracting teeth; and provided further, that none of the provisions of this act shall apply to regularly licensed physicians and surgeons in practice at or prior to the passage of this bill.

SEC. 7. Be it further enacted, That every person practicing dentistry in the State of Florida shall, within six months after the passage of this act, register his name, together with his post-office and the date of his certificate, in the office of the clerk of the circuit court of the county in which he practices, and shall, on the payment to such clerk of a fee of fifty cents, be entitled to receive from him a certificate of such registration.

SEC. 8. Be it further enacted, That every person practicing dentistry in the State of Florida at or prior to the passage of this bill shall be entitled to receive from the board of examiners a certificate to practice without undergoing an examination, on application by letter or otherwise; provided, that all such persons make application to said board within six months after the passage of this act.

SEC. 9. Be it further enacted, That all laws and parts of laws in conflict with this act be and they are hereby repealed.—*Cosmos.*

---

ARTICLE VII.

